# Assessing Risks and Innovating Traceability in Campania’s Illegal Mussel Sale: A One Health Perspective

**DOI:** 10.3390/foods14152672

**Published:** 2025-07-29

**Authors:** Valeria Vuoso, Attilio Mondelli, Carlotta Ceniti, Iolanda Venuti, Giorgio Ciardella, Yolande Thérèse Rose Proroga, Bruna Nisci, Rosa Luisa Ambrosio, Aniello Anastasio

**Affiliations:** 1 Department of Veterinary Medicine and Animal Production, University of Naples Federico II, 80137 Naples, Italy; valeria.vuoso@unina.it (V.V.); iolanda.venuti@unina.it (I.V.); anastasi@unina.it (A.A.); 2 Farzati S.P.A., 84040 Casal Velino, Salerno, Italy; attilio.mondelli@farzati.it (A.M.); giorgio.ciardella@farzati.it (G.C.); 3 Department of Health Sciences, University “Magna Græcia” of Catanzaro, 88100 Catanzaro, Italy; ceniti@unicz.it; 4 Department of Food Microbiology, Istituto Zooprofilattico Sperimentale del Mezzogiorno, 80055 Portici, Naples, Italy; yolande.proroga@cert.izsmportici.it; 5 Local Health Authority of Naples (A.S.L. Naples 1), c/o Presidio Frullone, 80145 Naples, Italy; bruna.nisci@aslnapoli1centro.it

**Keywords:** *Salmonella* Infantis, *Vibrio* spp., antimicrobial resistance, near-infrared spectroscopy, bivalve molluscs

## Abstract

The illegal sale of mussels is a persistent problem for food safety and public health in the Campania region, where bivalve molluscs are often sold without traceability, evading regulatory controls. In this study, ten batches of mussels seized from unauthorized vendors were analyzed to evaluate their microbiological safety and trace their geographical origin. High loads of *Escherichia coli*, exceeding European regulatory limits (Regulation (EC) No 2073/2005), were detected in all samples. In addition, *Salmonella* Infantis strains resistant to trimethoprim-sulfamethoxazole and azithromycin were isolated, raising further concerns about antimicrobial resistance. Of the 93 *Vibrio* isolates, identified as *V. alginolyticus* and *V. parahaemolyticus*, 37.63% showed multidrug resistance. Approximately 68.57% of the isolates were resistant to tetracyclines and cephalosporins. The presence of resistance to last-resort antibiotics such as carbapenems (11.43%) is particularly alarming. Near-infrared spectroscopy, combined with chemometric models, was used to obtain traceability information, attributing a presumed origin to the seized mussel samples. Of the ten samples, seven were attributed to the Phlegraean area. These findings have provided valuable insights, reinforcing the need for continuous and rigorous surveillance and the integration of innovative tools to ensure seafood safety and support One Health approaches.

## 1. Introduction

In 2022, global fisheries and aquaculture production reached 223.2 million tons, an increase of 4.4% compared to 2020. Of these, 130.9 million tons came from aquaculture, divided into 94.4 million tons of aquatic animals and 36.5 million tons of algae, with a total value of 313 billion dollars. Production is mainly concentrated in Asia, which alone represents 91.4% of the total, followed by both Latin America and the Caribbean (3.3%), and Europe (2.7%). In Europe, the main cultivated species includes molluscs (clams, mussels, oysters), salmonids, sea bream and sea bass, with significant attention to marine and coastal aquaculture, particularly developed in countries such as Norway, Spain, France and Italy [1]. Italy ranks fourth in the European Union for aquaculture production and tenth for fishing, with a total production of 130.000 tons, worth 499 million euros. Specifically, mussel production made up 46.5% of Italy’s total aquaculture output, valued at 61 million euros [2]. Mussels, a common name for various bivalve molluscs, include species like *Mytilus edulis* and *Mytilus galloprovincialis*, which are mainly produced and consumed in the European Union [1].

The significant development of the fishing sector, however, is also accompanied by a complex network of fraudulent practices. Seafood is among the most frequently adulterated food products globally, second only to oil, and the most affected among animal-based products. Accurately estimating the actual number of illegal activities in the fisheries sector remains difficult, as many go unnoticed. This undercounting occurs due to the lack of notification resulting from consumers’ limited awareness of safety regulations, which leads them not to perceive the risk [3].

According to the Ecomafia 2024 report from Legambiente [4], the fishing sector, in particular, in Campania, remains highly vulnerable to illicit activities, especially regarding the illegal fishing, distribution, and sale of molluscs without traceability, posing serious risks to public health. In particular, in 2023 over than 154.000 kg of fish products seized in Campania consisted of dates, crustaceans, and molluscs, often the result of illegal fishing or lacking necessary sanitary and documentary requirements [5]. These data underline the spread of a parallel, uncontrolled market that remains particularly difficult to dismantle, in part due to the complicity of consumers who purchase and consume products of unknown origin that do not comply with hygiene and safety standards. The main requirements for the marketing of live bivalve molluscs intended for human consumption are outlined in Regulation (EC) 853/2004 (Annex III, Section VII) [6] and Regulation (EC) No 2073/2005 [7], Regulation (EC) No 178/2002 (article 18) [8], Regulation (EU) No 1169/2011 [9], and Regulation (EU) No 1379/2013 [10].

Food business operators are not allowed to harvest live bivalve molluscs from areas not classified by the competent authority or deemed unsuitable for health reasons. These products must meet microbiological standards and be labeled according to EU legislation to ensure traceability. Nonetheless, illicit sales remain widespread and take various forms: molluscs of unknown origin that are removed from traceable packaging and placed into tanks with uncontrolled water, or sold by unauthorized vendors without proper storage. These practices increase the risk of microbiological contamination, especially during the summer, when high temperatures further compromise product quality. Such conditions not only endanger public health but also violate traceability requirements by erasing essential data on origin and handling. The illegal sale of unlabeled and unpackaged mussels by street vendors, along with inadequate storage practices, has been documented in Campania [5]. This issue is a concern for local authorities, highlighting the need for alternative methods to verify product origin and ensure consumer safety.

The lack of traceability and uncertainty regarding production conditions make it impossible to guarantee product security. Pathogenic bacteria may be hidden in these uncontrolled foods and pose a serious risk to public health, especially when consumed raw or undercooked, such as oysters and mussels [11]. Of particular concern is the presence of harmful bacteria that, in addition to carrying virulence and pathogenicity genes, may also have developed mechanisms of antibiotic resistance. The detection of antibiotic resistance genes in bacteria isolated from fish commonly consumed by humans is not an uncommon occurrence. The past decades have seen an alarming increase in the detection of antibiotic-resistant bacteria, exemplified by *Escherichia coli*, within marine environments. This phenomenon is likely correlated with the extensive use of antibiotics across human, agricultural, and aquaculture systems [12].

Therefore, in this context, the main objective of this study was to investigate both the presence and the antimicrobial resistance profile of potential pathogenic bacteria in mussels illegally sold in Naples (Campania region, Italy). Furthermore, to obtain useful information about the provenance of each sample, the Near-Infrared Spectroscopy (NIRS) was used, exploiting the potential of innovative technology. BluDev^®^ technology combines spectroscopy (Farzati Tech, Casal Velino, Italy), artificial intelligence (AI), and blockchain, with significant application potential in the agri-food sector.

## 2. Materials and Methods

### 2.1. Mussels Sampling

**Figure 1:**
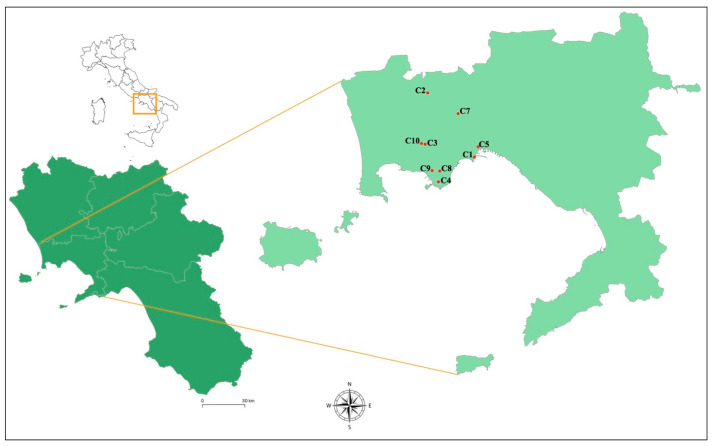
Geographic map with sampling points in the Naples province (Campania, Italy). The geographical distribution of sampling points was mapped using QGIS software, version 3.34. The geographical references relating to the coordinates (longitude and latitude; Table S1) of sampling points were transferred to the QGIS system to be merged with vectors of the Campania region.

In 2021, the Veterinary Services of the Local Health Authority of Naples (A.S.L. Naples 1) seized several kilos of mussels from 10 points of sale, as part of the repression activities carried out in various territorial districts (Figure 1). For each seizure point, several aliquots were prepared, each consisting of 10–13 individuals, which were necessary to reach 100 g of pulp and intravalvar liquid for microbiological analyses (120 samples analyzed in total), and a representative number of samples for NIRS analysis (10 samples per seizure point).

The samples were immediately and simultaneously transported to laboratories for analysis. Microbiological criteria were evaluated, according to the European Legislation (Reg. (UE) 2019/627 [13] and Reg. (EC) 2073/2005 [7]); therefore, *Salmonella* spp. research and *E. coli* enumeration were carried out. In addition, *Vibrio* species were researched and identified through a mass-spectroscopic approach. When *Salmonella* and *Vibrio* species were isolated, their antimicrobial resistance profiles were characterized. In conclusion, BluDev^®^ technology was used to define the specific origin of the mussels.

### 2.2. Culture Methods for Detecting Escherichia Coli and Vibro spp. in Mussels

Only live molluscs were considered for analysis; therefore, specimens with open or damaged shells were discarded. Molluscs with mud and scale on their valves were washed with running water and brushed to remove unwanted dirt. The byssus, when present, was cut with scissors before opening the valves with sterile briquettes. The intravalvar liquid and the pulp were placed inside a sterile bag until a weight of approximately 100 g was reached.

The detection and quantification of positive *Escherichia coli* β-glucuronidase was carried out according to the ISO 16649-3:2015 method, which is based on the most probable number (MPN) technique. The results were expressed as the most probable number of *E. coli* in 100 g of product (MPN/100 g). The MPN method provides an estimate of the most likely degree of contamination of a specific micro-organism within a food sample. For the calculation, per each dilution the number of positive tubes, confirmed on Tryptone Bile X-Glucuronide (TBX; OXOID) plates, was considered. The code thus obtained, indicative of the positive dilution tubes, was entered into the MPN calculator and the result obtained was expressed as *E. coli*/100 g. Negative results obtained were expressed as ≤18 *E. coli*/100 g.

For *Vibrio* spp., research and detection were performed following the ISO 21872-1:2017 method.

### 2.3. Vibrio Species Identification

Per each mussel batch, thirty colonies morphologically associated with *Vibrio* spp. were identified using MALDI-TOF/MS (MALDI Biotyper^®^ Sirius, Bruker, Billerica, MA, USA), adopting the direct colony identification method. In summary, colonies were duplicated and smeared onto a 96-point steel plate (Bruker Daltonics, Bremen, Germany). A 1 µL matrix solution, containing 10 mg/mL of α-cyano-4-hydroxycinnamic acid in acetonitrile (Sigma-Aldrich, Berlin, Germany), deionized water, and trifluoroacetic acid (50:47.5:2.5, [*v*/*v*/*v*]) was then applied. Bruker’s Bacterial Test Standard (BTS Bruker Daltonics) was used as a reference standard for mass calibration, and Flex Control 3.4 software (Bruker Daltonics, Bremen, Germany) was configured in the linear positive ion detection mode. Isolates were analyzed by matching their spectra with those in the Bruker MSP database (MBT Compass Library) using the Bruker Compass 1.4 software with default settings.

Identification score criteria were graded according to the manufacturer’s instructions: a score < 1.7 indicates unreliable identification, a score between 1.7 and 1.99 indicates probable genus identification, and a score ≥ 2 indicates a reliable identification of the species.

### 2.4. Vibrio Phenotypic Antimicrobial Susceptibility and Resistance Indexes

*Vibrio* colonies, identified by MALDI-TOF MS, were tested for antibiotic susceptibility using the Kirby–Bauer disc-diffusion method [14]. Specifically, the phenotypic antimicrobial susceptibility of each bacterial strain was evaluated towards the following antibiotics: Piperacillin-tazobactam (TZP 30-6 µg), Cefotaxime (CTX 5 µg), Ceftazidime (CAZ 10 µg), Meropenem (MRP 10 µg), Ciprofloxacin (CIP 5 µg), Levofloxacin (LEV 5 µg), Azithromycin (AZM 15 µg), Erythromycin (E 15 µg), Tetracycline (TE 30 µg), and Trimethoprim-sulfamethoxazole (SXT 1.25–23.75 µg). The protocols used agreed with the EUCAST guidelines, using bacterial inocula of 0.5 McFarland. Then, the discs were aseptically positioned on Muller–Hilton Agar (MHA, OXOID) plates and incubated at 37 °C for 18–24 h. The obtained inhibition zones were interpreted according to the EUCAST breakpoints [14]. Resistance to at least three antibiotics defines bacteria as multidrug resistant (MDR) [15].

In agreement with Ferri and colleagues (2024) [15], two phenotypic resistance indexes were determined. In more detail, the multiple antibiotic resistance (MAR) and the antibiotic resistance pattern (ARPA) indices were calculated considering all resistant bacteria, especially those bacteria that were MDR [16]. In particular, the *MAR* index was calculated as:

$${MAR}_{index}=a/b$$

where “*a*” is the total number of antibiotics to which each bacterium was found to be resistant, while “b” is the total number of tested antibiotics [15]. The threshold established for the MAR index was ≥0.2; this indicates significant health risks related to the uncontrolled use of antimicrobials, which contributes significantly to the spread of the phenomenon of antimicrobial resistance. To better frame this phenomenon, it is useful to consider the ARPA index [17], calculated as the ratio between the total resistance found and the total bacteria subjected to the antibiotic sensitivity study.

### 2.5. Salmonella Isolation and Phenotypic Antimicrobial Susceptibility

For rapid *Salmonella* spp. detection, a molecular approach was applied using the Real-Time PCR method (iQ-Check Salmonella II PCR Detection Kit, Bio-Rad, Hercules, CA, USA). The experimental protocol followed the manufacturer’s instructions.

To evaluate the antibiotic resistance profile of *Salmonella*, bacteria were isolated from positive samples using the culture-dependent research method in agreement with ISO 6579-1:2020. The Minimum Inhibitory Concentration (MIC) was determined through the broth microdilution method, according to ISO 20776-1. Singles colonies were transferred into 10 mL of Brain Heart Infusion (BHI) broth (OXOID), then incubated overnight at 37 °C to reach the desired inoculum concentrations. For a concentration of 0.5 McFarland, inocula were adjusted by carrying out dilutions with cation-adjusted Muller–Hilton broth (OXOID). Furthermore, further serial dilutions were performed to obtain the inoculum concentration to be used for tests (5 × 10^5^ CFU/mL). The MIC values were determined for thirteen antibiotics, which are listed below: Ceftazidime (CAZ), Ciprofloxacin (CIP), Chloramphenicol (CLO), Gentamicin (CN), Tetracycline (TE), Trimethoprim-Sulfamethoxazole (SXT), Tigecycline (TGC), Trimethoprim (TMP), Colistin Sulfate (COL), Nalidixic Acid (NAL), Ampicillin (AMP), Azithromycin (AZM), and Meropenem (MRP).

EUCAST breakpoints for bacteria belonging to the order Enterobacterales [14], were used to define *Salmonella* species as susceptible (S) or resistant (R).

### 2.6. Defining the Origin of Mussels Through the Application of an Implemented Near-Infrared Spectroscopy

Using Near-Infrared Spectroscopy (NIRS), Farzati has developed BluDev^®^ (Farzati Tech, Casal Velino, Italy), a proprietary technology based on three pillars: spectroscopy, artificial intelligence (AI), and blockchain technology. BluDev^®^ is a molecular sensor that facilitates qualitative and quantitative analyses directly on production lines or in mobile settings. By employing modern algorithms such as neural networks and chemometrics techniques, it precisely recognizes properties and characteristics of a matrix, creating its digital twin, also called a Digital Bio-Fingerprint (DBF). Spectra directly derived from matrix and all types of related data (raw materials information, traceability systems, production processes, supply chain management) are registered and notarized in a blockchain.

In this study, BluDev^®^ technology was used to obtain spectra for the ten batches of illegal mussels seized by the authorities, to compare them with reference standards and to establish the unknown origin of mussels. After external cleaning, all 100 seized specimens were opened and tested using the BluDev^®^ Next device (rev. 2022), working in a range of 640–1050 nm. Spectra were obtained by scanning internal mussels’ parts (gills, mantle, foot and palp) 10 times, moving the device each time to ensure coverage of the mussels’ entire valve areas. The spectra were acquired, taking care to keep the whole organs, avoiding scanning the shells. Therefore, a dataset of 1000 scans was obtained.

Standard reference spectra were obtained by scanning samples of known origin during several past inspection activities. In fact, due to characteristics of BluDev^®^ technology, it is necessary to collect many spectra from multiple sites to calibrate and evaluate a machine learning model able to discriminate the unknown origin of a sample. Spectra from mussels caught from the Phlegraean area, Greece, and Spain have been collected. Raw NIR spectra contain useful information, but also undesirable baseline shifts and non-linear scatter effects could be present: spectral pre-processing is required before conducting exploratory analysis. According to different studies [18,19], different pre-processing techniques were tested: Standard Normal Variate (SNV), Savitzky–Golay smoothing (SG), Multiplicative Scatter Corrections (MSC). The best model performances were obtained using the SNV technique.

### 2.7. Statistical Analysis

Before testing the model’s capacity to discriminate mussel origins, pre-processed spectra from seizures and standard references were used to perform a PCA (Principal Component Analysis) to evaluate potential clusters and data overlapping.

According to chemometrics’ best practices, the dataset was randomly divided into a Training Set (80%) and Validation Set (20%). Finally, the prediction of the mussels’ origins was assessed using a classification model with Train and Validation sets as input data. The construction, parameter tuning, and validation of the model were performed using the R package caret [20] and random forest as method for classification (with the number of trees set to 500).

## 3. Results and Discussion

### 3.1. Detection and Quantification of Target Bacteria in Compliance with Food Safety Criteria

The sale of mussels in conditions that do not comply with current European regulations is a practice that is unfortunately rooted in Campania, although it is increasingly rare. It involves the sale of molluscs operated by unlicensed sellers and/or molluscs without traceability and not in mesh bags (Table S1). Consumers are not aware of the hazards that may be hidden in these products, especially biological ones. When we refer to bivalve molluscs on the market for their retail sale, *E. coli* and *Salmonella* spp. must be studied to validate the compliance of the products with the European Regulation (EC) 2073/2005 [7]. In addition, emerging hazards, such as bacterial species or antimicrobial genes, must be paid attention to.

*E. coli* is a well-known indicator of fecal contamination, and its presence is notably significant when isolated in both food and environmental contexts. *E. coli* is widely recognized as a key indicator of fecal contamination and is used to assess the microbiological quality of seafood, particularly mussels [11]. The levels of fecal contamination in bivalve molluscs are measured using the MPN method; the analysis involves the entire body of the animal and the intravalvar liquid. Any excess above the limits established by the European legislation is considered a violation of food safety criteria, although *E. coli* is not a strictly pathogenic bacterium, and bivalve molluscs should be eaten well cooked. However, its presence in high quantities can represent a health problem, especially if related to the consumption of the product in unsafe ways such as undercooked or raw; furthermore, some *E. coli* species are responsible for foodborne disease (e.g., STEC) and the presence of *E. coli* is often correlated to the presence of other pathogens, both bacterial and viral.

In this context, the origin of the mussels from illegal stalls is not negligible, especially in a cultural context in which it is customary to consume this product undercooked. Although these products are not sold in compliance with the law and regulations, to correctly interpret the results it is necessary to classify them as “products placed on the market during their shelf life”. Based on these assumptions, it can be said that in all the samples analyzed, the levels of *E. coli* exceeded the limit established by Regulation (EC) 2073/2005 [7] (Table S2).

*Salmonella* spp. poses a notable risk considering the food safety criteria in Regulation (EC) 2073/2005 [7], which requires its absence in live molluscs sold on the market. According to the European Food Safety Authority (EFSA), in 2023, 10.59% of outbreaks linked to fish and fishery products consumption were attributed to *Salmonella* spp., including multidrug-resistant (MDR) strains, escalating its threat [21]. The EFSA report, related to the years 2020/2021, reveals that resistance to ampicillin, sulfonamides, and tetracyclines was noted at elevated levels in *Salmonella* spp. isolated from humans in 2021, with a range from moderate to very high in isolates from food-producing animals and poultry carcasses. Multidrug resistance was generally high among *Salmonella* spp. reported in human cases in the EU (22.5%). Similarly, MDR has been observed at moderate-to-very high levels in *Salmonella* spp. recovered from turkey and broiler carcasses (19.1% and 53.6%, respectively) and at high levels in fattening pigs (39.1%), calves (30.4%), fattening turkeys (41.7%), and broilers for fattening (44%). Resistance to multiple antibiotics has been observed in *S*. Infantis and other serotypes, bolstering their epidemiological prominence [22].

**Figure 2:**
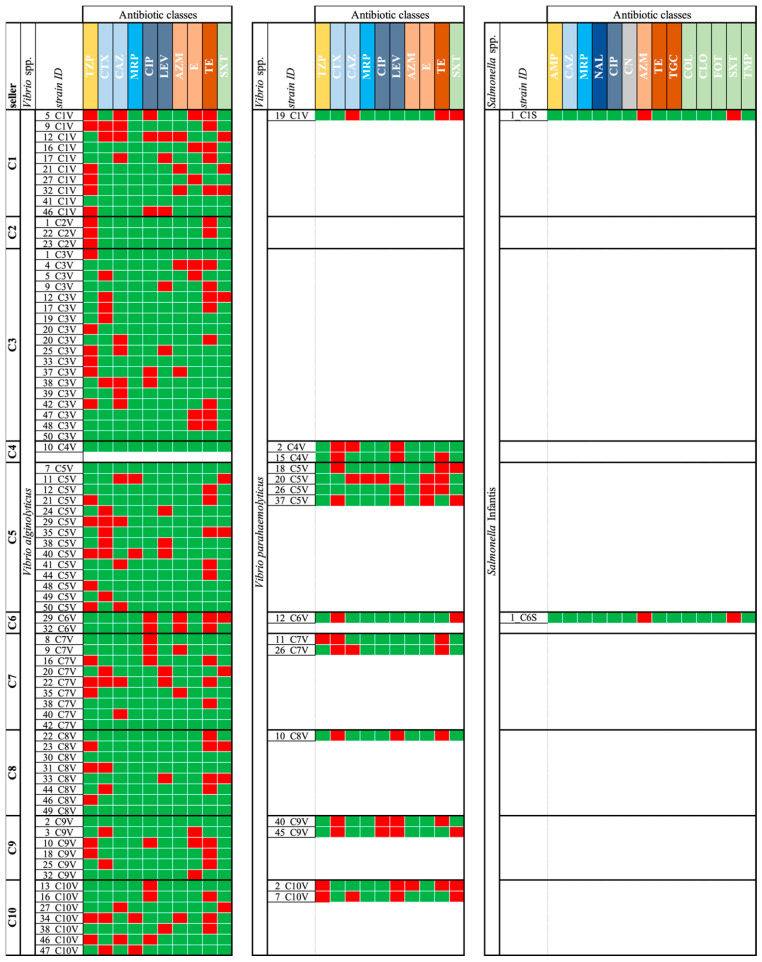
Schematic representation of the phenotypic resistance profiles of *V. alginolyticus*, *V. parahaemolyticus* and *Salmonella* Infantis bacterial strains isolated from the ten seized samples (C1–C10). Red squares indicate resistant bacteria, while green squares indicate susceptibility. AMP: Ampicillin, AZM: Azithromycin, CAZ: Ceftazidime, CIP: Ciprofloxacin, CLO: Chloramphenicol, COL: Colistin Sulfate, CN: Gentamicin, CTX: Cefotaxime, E: Erythromycin, LEV: Levofloxacin, MRP: Meropenem, NAL: Nalidixic Acid, SXT: Trimethoprim-Sulfamethoxazole, TE: Tetracycline, TGC: Tigecycline, TMP: Trimethoprim, and TZP: Piperacillin-tazobactam.

Our analyses aimed at detecting *Salmonella* spp. indeed unveiled the presence of *S*. Infantis in two samples. Additionally, the isolated strains exhibited notable resistance to two antibiotics: trimethoprim-sulfamethoxazole and azithromycin (Figure 2). While in the European Union territory *S*. Enteritidis is the most common serovars responsible for non-typhoidal salmonellosis, *S.* Infantis is the most isolated in foods in Italy (approximately 47.55% in 2022 [23], and the first serovar isolated from the “broiler” source (96.1%) in 2023 [21].

### 3.2. Vibrio spp., Emerging Pathogenic Species

The dynamism of EU food legislation is driven by the emergence of new global public health challenges. Analyzing new risks is necessary to identify the critical concentration limits above which food containing a hazard could be harmful to the consumer. New hazards and challenges are reported by both scientific communities, through their publications, and by Food Business Operators and Competent Authorities, through self-control and official control activities. In this scenario, some *Vibrio* species are gaining ground among emerging bacterial pathogens due to their role in causing foodborne disease worldwide. The main pathogen vehicle is seafood, involved in 32 cases of foodborne outbreaks (FBOs) with strong and weak evidence, especially per the presence of *V. parahaemolyticus* (30/32). This bacterium was responsible for 221 human cases and 57 hospitalizations. The list of virulence and pathogenicity genes is also evolving; until a few years ago, the genes encoding the hemolysins TDH and TRH (thermostable direct hemolysin, TDH, or TDH-related hemolysin, TRH) were the only ones considered. However, a small portion of *V. parahaemolyticus* isolated from those human cases (~10%) did not show positivity for these genes, highlighting the plausible presence of other virulence factors. *V. alginolyticus* may also occasionally be involved in human cases of disease, especially in the most susceptible target individuals (immunocompromised) [24].

The challenges related to this bacterial genus are more than those mentioned above; for instance, depuration, the standard method for depurating bivalve molluscs from coliforms (*E. coli*, in particular; Regulation UE 2019/627 [13]), is not properly effective in removing and cleaning filter-feeding organisms from *Vibrio* [25]. Food business operators should control and monitor all the variables that can influence the depuration of organisms from these bacteria, such as water temperature, salinity, and pH, and the dwell time of molluscs in water. European legislation does not set the hours or days necessary for depuration, and food business operators refer to scientific data that confirm the validity of their applied methods. However, until the legislators consider *Vibrio*, the methods will be evaluated on microorganisms such as *E. coli*, which are more sensitive to depuration processes. Bivalve molluscs could be vehicles of these bacteria and, therefore, of the antibiotic-resistant genes (ARGs) commonly present in their genome [26].

In this study, particular attention was paid to the *Vibrio* genus, investigating their presence in 10 batches of mussels. In each mussel batch, characteristic colonies of *Vibrio* were isolated; identification with MALDI-TOF confirmed our suspicions and highlighted the presence of potentially dangerous enteropathogenic species. In more detail, out of the total of 300 colonies subjected to identification (30 characteristic colonies per mussel batch), 177 colonies were identified at the species level (58%). The most frequently isolated bacterial species were *Vibrio alginolyticus* (44.07%), *Shewanella putrefaciens* (24.29%), and *Proteus mirabilis* (19.77%) (Figure S1). It is not uncommon to identify genera other than *Vibrio* that are capable of growing on TCBS agar [27].

As shown in Figure S1, the main bacterial species identified was *V. alginolyticus*. In humans, this bacterium is usually a vehicle of extraintestinal infections, mainly skin infections resulting from contact with seawater. However, the gastrointestinal pathogenic potential of *V. alginolyticus* in humans has also been recognized, even capable of causing mortality in immunocompromised patients [28]. The other *Vibrio* species isolated was *V. parahaemolyticus*, a ubiquitous bacterium, with a global distribution, primarily inhabiting coastal marine ecosystems. Its prevalence poses a significant food safety concern, particularly in marine-derived products from temperate or tropical waters, including bivalve molluscs, crustaceans, and fish. The ingestion of raw or contaminated seafood harboring *V. parahaemolyticus* can lead to acute gastroenteritis, manifested by symptoms such as diarrhea, vomiting, and abdominal cramps. Consequently, *V. parahaemolyticus* stands as the predominant causative agent of seafood-associated gastroenteritis worldwide.

Despite the implementation of diverse preservation methodologies, *V. parahaemolyticus* exhibits resilience within seafood matrices, contributing to its sustained prevalence. This persistence is notably accentuated by the emergence of specific serotypes, notably those carrying the *tdh* gene [29]. However, it is not only virulence and pathogenicity genes that are cause of concern, but also those related to antibiotic resistance. When a strain is equipped with them, it not only acquires resistance to a certain antibiotic, but becomes a carrier and donor of the same genes to other bacteria.

**Figure 3:**
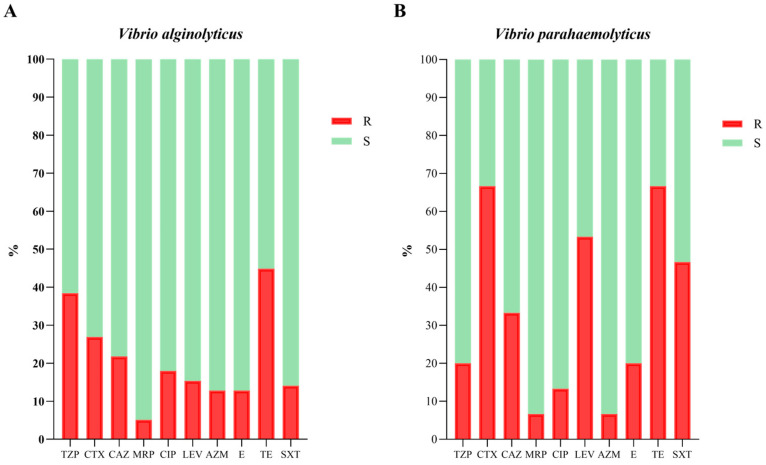
Percentage of antibiotic resistance (R) and sensitivity (S) to antibiotics for *V. alginolyticus* (**A**) and *V. parahaemolyticus* (**B**) strains. TZP: Piperacillin-tazobactam; CTX: Cefotaxime; CAZ: Ceftazidime; MRP: Meropenem; CIP: Ciprofloxacin; LEV: Levofloxacin; AZM: Azithromycin; E: Erythromycin; TE: Tetracycline; and SXT: Trimethoprim-Sulfamethoxazole.

Aware of the importance and need to contain the spread of ARGs and, therefore, of MDR bacteria, a phenotypic evaluation of the antibiotic resistance profile of *Vibrio* strains isolated from the mussels was carried out. In particular, a total of 93 *Vibrio* strains were subjected to antimicrobial susceptibility testing toward ten antibiotics. After incubation, the inhibition zones were recorded for each strain and data were analyzed according to EUCAST guidelines [14]. The specific antibiotic resistance profile of each *Vibrio* isolated in this study is shown in Figure 2. No strain showed resistance to more than six antibiotics. In more detail, 1.08% (1/93 strains), 4.30% (4/93), and 10.75% (10/93) showed resistance to six, five, and four antibiotics, respectively; 27.96% (26/93), 29.03% (27/93) and 18.28% (17/93) were resistant to three, two, and one antibiotic, respectively; and finally, only 8.60% (8/93) were sensitive to all tested antibiotics (10/10). Overall, the highest percentage of resistance was detected against TE (48.39%, 45/93 strains), followed by resistance to TZP (35.48%, 33/93), CTX (33.33%, 31/93), CAZ (23.66%, 22/93), and LEV (22.58%, 21/93). On the other hand, most of the *Vibrio* strains were found to be susceptible to MRP (94.62%, 88/93), AZM (88.17%, 82/93) and E (86.02%, 80/93). Analyzing the data by species, the results were slightly different (Figure 3). Higher resistance rates have been described for *V. parahaemolyticus* strains. However, it is worth noting that fewer strains belonging to the *parahaemolyticus* species were isolated in the present study than those belonging to the *alginolyticus* species. For these reasons, the percentages were calculated on different numbers of strains (78 strains of *V. a*. and 15 strains of *V. p*.) and this may influence the calculation of the percentages that are difficult to compare with each other. In any case, for both species the highest resistance rate was against TE (66.67% and 44.87% for *V. p*. and *V. a*., respectively), while the lowest was against MRP (6.67% and 5.13% for *V. p*. and *V. a.*, respectively). Meanwhile, the principal differences were in the resistance to CTX, LEV, and SXT, with *V. parahaemolyticus* strains showing higher resistance (Figure 3B).

**Figure 4:**
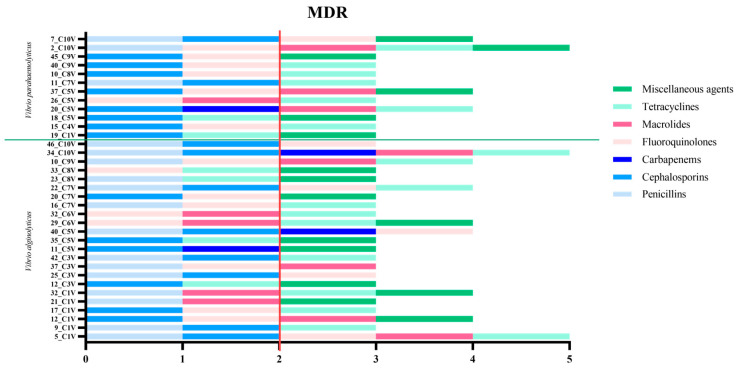
Representation of the multidrug resistance profile of the 35 *Vibrio* bacterial isolates in relation to antibiotic classes. Each horizontal bar represents a single isolate and the antibiotic classes to which it showed phenotypic resistance. Antibiotic classes are color-coded. The red vertical line indicates the limit beyond which the isolates are considered MDR, as they are resistant to three or more different antibiotic classes.

In summary, the analyses performed revealed a widespread and varied presence of antibiotic resistance which made the strains resistant to multiple classes of antibiotics (Figure S2). In particular, 37.63% (35/93 *Vibrio* strains) of the strains examined were found to be multidrug-resistant (Figure 4), meaning resistant to at least three different classes of antibiotics. It is worth underlining that almost all *V. parahaemolyticus* strains were multidrug-resistant (80%, 12/15 strains); lower percentages were obtained for *V. alginolyticus* (29.49%, 23/78 strains).

Multidrug-resistant strains showed resistance to several classes of antibiotics, with the highest rates observed for tetracyclines and cephalosporins, both affecting 68.57% of *Vibrio* isolates. High levels of resistance were also recorded for fluoroquinolones (62.86%), followed by penicillins (48.57%), miscellaneous agents (45.71%), and macrolides (37.14%). Particularly concerning is the resistance to carbapenems, identified in 11.43% of the resistant strains. Although relatively low, this finding is significant given the critical role of carbapenems as last-resort antibiotics. Their reduced efficacy, even in a small proportion of isolates, raises serious concerns about treatment options and the potential for further dissemination of resistance.

Recent studies suggest that multidrug resistance in vibrios and other enteric pathogenic bacteria is mainly attributable to horizontal gene transfer. This process involves the movement of mobile genetic elements, such as plasmids, transposons, and integrons, between different bacteria, regardless of their phylogenetic relationship. These genetic elements are highly dynamic and facilitate the rapid transfer of resistance genes, contributing to the spread of antibiotic resistance [30]. These horizontal transfer mechanisms could explain the isolation of several MDR strains from the same sample, as well as the simultaneous presence of resistance to trimethoprim-sulfamethoxazole and azithromycin in *Salmonella* and *Vibrio* strains isolated from samples C1 and C6.

A more in-depth analysis of antibiotic resistance patterns further highlights the critical situation regarding multidrug resistance among *Vibrio* isolates. The Multiple Antibiotic Resistance (MAR) index for *V. parahaemolyticus* was particularly high (0.34 ± 0.07), exceeding the critical threshold of 0.2 across all isolates, suggesting that these strains likely originate from environments with intense antibiotic pressure. *V. alginolyticus* showed greater variability (mean = 0.21 ± 0.13; min = 0; max = 0.6), indicating a mixed population of resistant and susceptible strains. This variability is due both to the greater number of *V. alginolyticus* strains isolated in this study and to the variability among the seized mussel samples. Indeed, referring to Figure S3 it is possible to note the different placement of the 10 samples with respect to the impact of antibiotic use and persistence in the environment. Overall, in fact, the MAR value was higher for the strains isolated from samples C1, C6, and C10 (0.32 ± 0.05, 0.30 ± 0.06, and 0.29 ± 0.05, respectively; mean ± error standard), followed by C7, C9, and C5 (0.22 ± 0.07, 0.23 ± 0.05, and 0.24 ± 0.08, respectively; mean ± error standard). Aggregating data from all *Vibrio* isolates, the mean MAR index was also above the critical threshold (0.23 ± 0.13), again highlighting the importance of the observed antibiotic resistance profiles and raising concerns about the ability to spread these types of resistance.

Furthermore, the antibiotic resistance pattern (ARPA) index for the 93 *Vibrio* strains was 2.31. Since this index is calculated as the ratio between the total resistances found and the total bacteria subjected to the antibiotic sensitivity study, it is possible to state that each *Vibrio* strain was resistant, on average, to more than two antibiotics. More specifically, the index was 2.10 for *V. alginolyticus* strains and 3.40 for *V. parahaemolyticus* strains.

The values of the MAR and ARPA indices underscore the severity of antibiotic resistance in these *Vibrio* strains, highlighting the likelihood that the mussels were exposed to environments with high antibiotic pressure.

### 3.3. Mussels’ Traceability

It is well known that NIRS is a widely used technique in several fields, like food analysis [31]. Using the infrared part of the spectrum (about 800–2500 nm), it examines how electromagnetic waves interact with a sample, exploring its physical–chemical properties. The underlying principle of NIRS involves the interaction of infrared radiation with molecular vibrations. When molecules absorb energy at specific wavelengths within the near-infrared spectrum, they undergo a transition to an excited vibrational state. Subsequent relaxation to the ground state results in the emission of infrared light, which is quantitatively measured by a specialized detector. This process yields a unique absorption profile characteristic of the analyzed sample’s distinct physicochemical properties. Physically, the NIR radiation primarily influences combination bands and overtones, which are mostly linked to C-C, O-H, and N-H bonds. The resulting signal is generally weak, but highly characteristic. Compared to traditional chemical analysis methods, NIRS stands out for its speed and simplicity. Measurements typically take only 5–10 s and deliver a high accuracy and reproducibility. Crucially, NIRS requires no preliminary sample preparation or specialized technical training for the operator, making it a highly accessible tool. As reported by [32], NIRS provides valuable information on the chemical composition and physical properties of samples, as demonstrated in studies on mussel tissues and shells, including the bioaccumulation of inorganic elements. These characteristics are significantly influenced by factors such as the mussels’ geographical origin, size, and spatial distribution. Consequently, analyzing trace element levels in samples through NIRS can help differentiate between various geographical origins.

**Figure 5:**
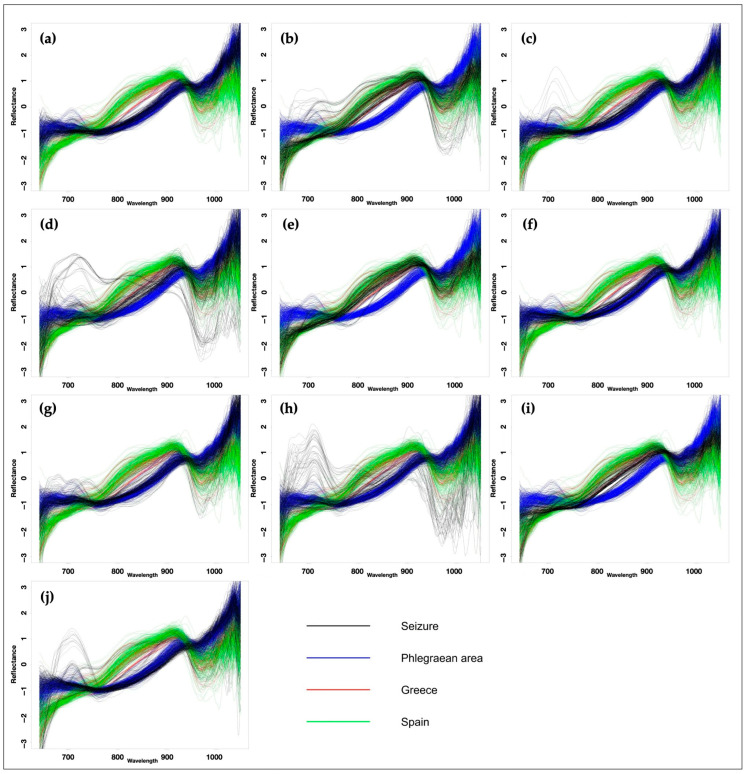
NIR spectra of the seized samples (black) compared with the calibration spectra used as reference, i.e., mussels of Phlegraean (blue), Greek (red) and Spanish (green) origin. (**a**): Seized sample C1; (**b**): seized sample C2; (**c**): seized sample C3; (**d**): seized sample C4; (**e**): seized sample C5; (**f**): seized sample C6; (**g**): seized sample C7; (**h**): seized sample C8; (**i**): seized sample C9; and (**j**): seized sample C10.

In this study, for each seized sample the spectra were overlaid and compared with the reference standards, i.e., with calibration spectra of mussels of Phlegraean origin, Greek origin, and Spanish origin (Figure 5). The hypothesized match was finally attributed to the origin that showed the highest percentage among the three loaded into the dataset (detailed information is reported as supplementary data, in Table S3).

The random forest model classified ten seized samples based on their correspondence to standard references. Therefore, by superimposing the spectra of the samples with those of the reference standards, it was possible to state that seven samples (seized samples 1, 3, 4, 6, 7, 8 and 10) should have Phlegraean origin, due to the highest percentage of overlap with the respective reference spectra (Figure 5); in contrast, in three samples the highest percentage of overlap with the reference spectra of Greek mussels suggests a Greek origin (seized samples 2, 5 and 9). Percentages of overlap seem to suggest that no sample has a Spanish origin. Regarding the percentage of correspondence, in four cases (seized samples 1, 3, 6 and 7) a very strict correspondence (>90%) with the reference spectra of Phlegraean origin was reported; in particular, only seized sample 1 (C1) showed 100% correspondence. On the other hand, seized samples 2 and 5 showed the weakest correspondence with the standard references, with a 59% and 61.5% match, respectively (Table S3). The *Random Forest model* employed in this study classifies seized samples based on the three geographical origins established in the training dataset. The percentage assignment to each class is derived from the affinity of the sampled Near-Infrared (NIR) spectra. It was observed that certain groups, specifically C2, C4, and C5, exhibited relatively low affinity percentages, approximating 60%. This comparatively modest value may stem from several contributing factors: potential sampling errors—given the inherent difficulty of obtaining consistent measurements from the curved shells of molluscs—or the poorly defined boundaries among the three designated origin groups. Future implementations will focus on two key improvements: refining the sampling methodologies and substantially increasing the number of spectra utilized in the construction of the training dataset.

**Figure 6:**
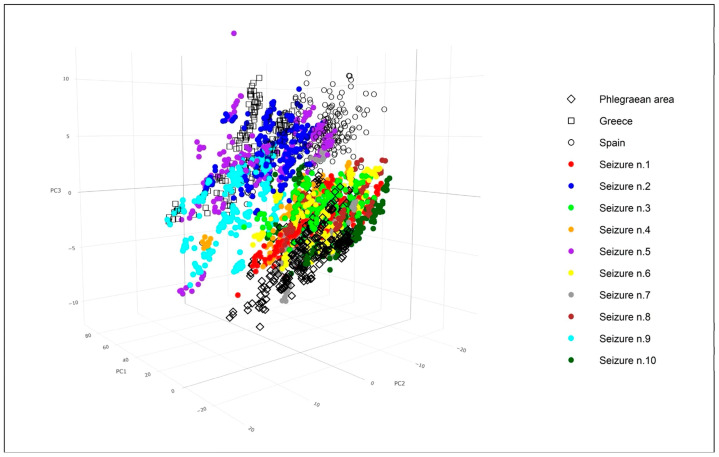
Three-dimensional PCA scatter plots of the first three principal components.

A Principal Component Analysis (PCA) was performed to evaluate the spectral overlap between seized samples and established reference standards. Figure 6, which visualizes the PCA scores for the first three components, reveals that PC1, PC2, and PC3 account for 72.7%, 14.4%, and 4.5% of the total explained variance, respectively.

As evident from Figure 6, the standard reference samples tended to group into three distinct clusters, corresponding to their three different geographical origins. Notably, the spectra of the seized samples exhibited varying degrees of overlap within the same three-dimensional space as these standard references. The degree of overlap between the different clusters is a critical indicator of how effectively the PCA embedding distinguishes them. Significant overlap signifies similarity, while clear separation implies distinct underlying characteristics. Looking at the standard references, the “Phlegraean area” (black diamonds) and “Greece” (black squares) data points form relatively distinct clusters, showing good separation from each other and from most of the seized sample categories. In contrast, the “Spain” points (black circles) are notably more scattered, indicating greater variability within this reference group.

Among the seized samples, there is a discernible attempt to differentiate between various types. Some seized samples form relatively compact clusters, suggesting internal homogeneity. For instance, samples 1, 3, 6, 7, 8, and 10 visually cluster together in the lower-right quadrant of the plot.

There was notable overlap between the “Phlegraean area” reference samples and several seized samples (specifically C1, C3, and C6). This overlap could suggest shared underlying characteristics or a less definitive distinction between these particular seized samples and the Phlegraean origin when based solely on these principal components. Conversely, “Phlegraean area” and “Greece” showed better separation, as do some pairwise comparisons between seized samples (e.g., C1 versus C9). This improved separation suggests that these groups possess more distinctive features captured by the principal components.

A more detailed examination reveals that seized samples 1, 3, 4, 6, 7, 8, and 10 exhibited overlap within the three-dimensional space corresponding to the Phlegraean standard references. Conversely, seized sample 9 demonstrated an overlap with the Greek standard references. Lastly, seized samples 2 and 5 were observed to share common three-dimensional space with both the Greek and Spanish standard references.

The 3D PCA plot in Figure 6 effectively visualizes the complex relationships and distinctions among the various data points, immediately highlighting both well-separated clusters and significant areas of overlap based on their underlying spectral characteristics. This figure also provides direct insights into the geographical origins of the seized samples. Consequently, these findings lead to the hypothesis that most of the seized mussels are derived from local aquaculture operations.

The application of NIRS technology, in conjunction with robust decision-making models and blockchain technology [33], emerges as a reliable methodology for ensuring the quality and traceability of mussels, thus preserving the interests of both consumers and producers. Beyond regulatory applications, this technique could also be adopted by food industry operators seeking to authenticate and certify product origin, thus valorizing their production. Concurrently, blockchain technology would ensure unassailable transparency and legality, thereby empowering consumers with informed and confident choices. The BluDev^®^ technology, employed in this investigation, was specifically engineered to fulfill these needs, providing not only expeditious support for origin identification but also the capacity to render information immutable and transparent for end-users.

## 4. Conclusions

The results of this study highlight significant health risks associated with the illegal sale of mussels in the Campania region. All batches analyzed showed non-compliance with European microbiological criteria, including levels of *E. coli* well above the permitted thresholds and the presence of antibiotic-resistant *Salmonella* Infantis in two samples. Furthermore, the detection of high percentages of multidrug-resistant *Vibrio* spp. (over 35%), in particular *V. parahaemolyticus*, raises further concerns about the increasingly relevant role of seafood in the spread of antimicrobial resistance.

The detection of resistance traits in pathogenic and non-pathogenic bacteria in the marine environment is a crucial indicator to monitor the spread of antimicrobial resistance and understand the close interconnection between human health, animal health, environmental integrity, and food safety.

From a traceability perspective, the application of the BluDev^®^ technology that exploits near-infrared spectroscopy has proven to be a practical, portable, powerful, and innovative tool, allowing reliable identification of the origin of mussels even in the absence of documentation. Most of the samples were traced back to the Phlegraean area, suggesting the involvement of local aquaculture activities.

Overall, these results highlight the urgent need for coordinated efforts involving public health authorities, veterinary services and food business operators to monitor, prevent and combat illegal practices in the fisheries sector. Adopting a One Health approach is essential to address the interconnected risks of environmental contamination, antimicrobial resistance and illegal food sales. A multidisciplinary strategy that promotes consumer awareness, regulatory compliance and the integration of innovative technologies such as NIRS is essential to improve food safety, environmental sustainability and public health, while helping to protect responsible producers and ensure transparency along the entire food supply chain.

## 5. Patents

Trademark Registration Number 302019000045240, filed on 1 July 2019.

SIAE (Italian Society of Authors and Publishers) registration, with number D000013706 and progressive number D000014588, made on 19 June 2020.

## Data Availability

The raw data supporting the conclusions of this article will be made available by the authors on request.

## Supplementary Materials

The following supporting information can be downloaded at: https://www.mdpi.com/article/10.3390/foods14152672/s1, Table S1. Coordinates of the seizure points for each sample (latitude and longitude). Table S2. Concentration of E. coli in the ten seized samples (from C1 to C10) and expressed in MPN/100 g. Table S3. Percentage of correspondence with reference spectra for each seized sample; Figure S1: Percentages of bacterial species isolated from 10 batches of mussels. Figure S2. Representation of resistance profiles of each Vibrio bacterial isolate in relation to antibiotic classes. Each horizontal bar represents a single isolate and the antibiotic classes to which it showed phenotypic resistance. Figure S3. Average MAR index values (± error standard) calculated for Vibrio strains belonging to each single seized sample.

## Abbreviations

The following abbreviations are used in this manuscript:

EUCAST	European Committee on Antimicrobial Susceptibility Testing
NIRS	Near-Infrared Spectroscopy
AI	Artificial Intelligence
A.S.L.	Local Health Authority
QGIS	Quantum Geographic Information System
MPN	Most Probable Number
MDR	Multidrug Resistant
MAR	Multiple Antibiotic Resistance
ARPA	Antibiotic Resistance Pattern
MIC	Minimum Inhibitory Concentration
DBF	Digital Bio-Fingerprint
SNV	Standard Normal Variate
SG	Savitzky–Golay smoothing
MSC	Multiplicative Scatter Corrections
PCA	Principal Component Analysis
FBO	Foodborne Outbreak
ARGs	Antibiotic Resistance Genes
AMP	Ampicillin
AZM	Azithromycin
CAZ	Ceftazidime
CIP	Ciprofloxacin
CLO	Chloramphenicol
COL	Colistin Sulfate
CN	Gentamicin
CTX	Cefotaxime
E	Erythromycin
LEV	Levofloxacin
MRP	Meropenem
NAL	Nalidixic Acid
SXT	Trimethoprim-Sulfamethoxazole
TE	Tetracycline
TGC	Tigecycline
TMP	Trimethoprim
TZP	Piperacillin-tazobactam

## References

[1] Food and Agriculture Organization of the United Nations (FAO) (2024). The State of World Fisheries and Aquaculture 2024.

[2] European Market Observatory for Fisheries and Aquaculture Products (EUMOFA) (2025). Country Profile: Italy—Italy in the World and in the EU.

[3] Armani A., D’Amico P., Guidi A., Cenci Goga B. (2018). Le frodi nel comparto ittico. Ispezione e Controllo Degli Alimenti.

[4] Osservatorio Nazionale Ambiente e Legalità (2024). Ecomafia 2024: Le Storie E I Numeri Della Criminalità Ambientale.

[5] Legambiente (2024). Mare Monstrum 2024: Abusivismo Edilizio, Inquinamento, Pesca Illegale. I Numeri E Le Storie Dell′Aggressione Criminale Alle Coste E Al Mare Del Nostro Paese.

[6] Council of the European Union (2004). Regulation (EC) No 853/2004 of the European Parliament and of the Council of 29 April 2004 laying down specific hygiene rules for food of animal origin. Off. J. Eur. Union.

[7] Council of the European Union (2005). Commission Regulation (EC) No 2073/2005 of 15 November 2005 on microbiological criteria for foodstuffs. Off. J. Eur. Union.

[8] Council of the European Union (2002). Regulation (Ec) No 178/2002 of The European Parliament And of The Council of 28 January 2002 laying down the general principles and requirements of food law, establishing the European Food Safety Authority and laying down procedures in matters of food safety. Off. J. Eur. Communities.

[9] Council of the European Union (2011). Regulation (EU) No 1169/2011 of the European Parliament and of the Council of 25 October 2011 on the provision of food information to consumers, amending Regulations (EC) No 1924/2006 and (EC) No 1925/2006 and repealing several directives. Off. J. Eur. Union.

[10] Council of the European Union (2013). Regulation (EU) No 1379/2013 of the European Parliament and of the Council of 11 December 2013 on the common organisation of the markets in fishery and aquaculture products, amending Council Regulations (EC) No 1184/2006 and (EC) No 1224/2009 and repealing Council Regulation (EC) No 104/2000. Off. J. Eur. Union.

[11] Yibar A., Saticioglu I.B., Ajmi N., Duman M. (2024). Molecular Characterization and Antibacterial Resistance Determination of Escherichia Coli Isolated from Fresh Raw Mussels and Ready-to-Eat Stuffed Mussels: A Major Public Health Concern. Pathogens.

[12] Mancini M.E., Alessiani A., Donatiello A., Didonna A., D’Attoli L., Faleo S., Occhiochiuso G., Carella F., Di Taranto P., Pace L. (2023). Systematic Survey of *Vibrio* spp. and *Salmonella* spp. in Bivalve Shellfish in Apulia Region (Italy): Prevalence and Antimicrobial Resistance. Microorganisms.

[13] Council of the European Union (2019). Commission Implementing Regulation (EU) 2019/627 of 15 March 2019 laying down uniform practical arrangements for the performance of official controls on products of animal origin intended for human consumption. Off. J. Eur. Union.

[14] European Committee on Antimicrobial Susceptibility Testing (EUCAST) (2025). Breakpoint Tables for Interpretation of MICs and Zone Diameters, Version 15.0. https://www.eucast.org/clinical_breakpoints/.

[15] Ferri G., Olivieri V., Olivastri A., Pennisi L., Vergara A. (2024). Multidrug Resistant *Vibrio* Spp. Identified from Mussels Farmed for Human Consumption in Central Italy. J. Appl. Microbiol..

[16] Adesiyan I.M., Bisi-Johnson M.A., Okoh A.I. (2022). Incidence of Antibiotic Resistance Genotypes of *Vibrio* Species Recovered from Selected Freshwaters in Southwest Nigeria. Sci. Rep..

[17] Deng Y., Xu L., Chen H., Liu S., Guo Z., Cheng C., Ma H., Feng J. (2020). Prevalence, Virulence Genes, and Antimicrobial Resistance of Vibrio Species Isolated from Diseased Marine Fish in South China. Sci. Rep..

[18] Riu J., Gorla G., Chakif D., Boqué R., Giussani B. (2020). Rapid Analysis of Milk Using Low-Cost Pocket-Size NIR Spectrometers and Multivariate Analysis. Foods.

[19] Modroño S., Soldado A., Martínez-Fernández A., De La Roza-Delgado B. (2017). Handheld NIRS Sensors for Routine Compound Feed Quality Control: Real Time Analysis and Field Monitoring. Talanta.

[20] Kuhn M. (2008). Building predictive models in R using the caret package. J. Stat. Softw..

[21] EFSA, ECDC (2024). The European Union One Health 2023 Zoonoses Report. EFSA J..

[22] Montone A.M.I., Cutarelli A., Peruzy M.F., La Tela I., Brunetti R., Pirofalo M.G., Folliero V., Balestrieri A., Murru N., Capuano F. (2023). Antimicrobial Resistance and Genomic Characterization of Salmonella Infantis from Different Sources. Int. J. Mol. Sci..

[23] Centro di Referenza Nazionale per le Salmonellosi, Istituto Zooprofilattico Sperimentale delle Venezie (2024). Enter-Vet Report 2022.

[24] EFSA Panel on Biological Hazards (BIOHAZ) (2024). Scientific opinion on public health aspects of *Vibrio* spp. related to the consumption of seafood in the EU. EFSA J..

[25] FAO, WHO (2020). Risk Assessment Tools for Vibrio Parahaemolyticus and Vibrio Vulnificus Associated with Seafood.

[26] Fang G.-Y., Liu X.-Q., Mu X.-J., Huang B.-W., Jiang Y.-J. (2023). Distinct Increase in Antimicrobial Resistance Genes among *Vibrio Parahaemolyticus* in Recent Decades Worldwide. Chemosphere.

[27] Shikongo-Nambabi M.N.N., Chimwamurombe P.M., Venter S.N. (2012). Identification of Putative *Vibrio* Species Isolated from Processed Marine Fish Using Thiosulphate-Citrate-Bile-Sucrose (TCBS) Agar. Br. Biotechnol. J..

[28] Mustapha S., Ennaji M.M., Cohen N. (2013). *Vibrio alginolyticus*: An emerging pathogen of foodborne diseases. Int. J. Sci. Technol..

[29] Nelapati S., Nelapati K., Chinnam B.K. (2012). *Vibrio parahaemolyticus*—An emerging foodborne pathogen: A review. Vet. World.

[30] Dutta D., Kaushik A., Kumar D., Bag S. (2021). Foodborne Pathogenic Vibrios: Antimicrobial Resistance. Front. Microbiol..

[31] Pasquini C. (2018). Near Infrared Spectroscopy: A Mature Analytical Technique with New Perspectives—A Review. Anal. Chim. Acta.

[32] Puleo S., Di Monaco R., Luca Langellotti A., Masi P. (2022). The Origin of Mussels (*Mytilus galloprovincialis*): NIRS Explanatory Identification and the Effect on Consumers. Food Chem..

[33] Botta V., Fusco L., Mondelli A., Visconti I. Secure blockchain-based supply chain management with verifiable digital twins. Proceedings of the 2023 ACM Conference on Information Technology for Social Good.

