# Supplementary material for: Assessing Risks and Innovating Traceability in Campania’s Illegal Mussel Sale: A One Health Perspective
Source: Foods. 2025 Jul 29;14(15):2672. doi: 10.3390/foods14152672 (PMC12346825; doi:10.3390/foods14152672)
Supplement: Supplementary file 1 [file foods-14-02672-s001.zip › foods-3750535-supplementary.pdf]

Supplementary data

Table S1. Coordinates of the seizure points for each sample (latitude and longitude)

Sample ID	Type	Seizure Location	Coordinates (lat, long)
**C1**	Submerged Rows	Porto Napoli	40.83301172677624, 14.252120063263943
**C2**	Unlicensed Street Vendor	Corso Campano	40.9252483045508, 14.174325841120027
**C3**	Unlicensed Street Vendor	Via Nelson Mandela	40.85495104540085, 14.164319439263943
**C4**	Unlicensed Street Vendor	Viale Virgilio	40.8021361784392, 14.184242268096233
**C5**	Unlicensed Street Vendor	Porto Napoli	40.84711697472475, 14.26030956825493
**C6**	Unlicensed Street Vendor	Via Cesare Rosaroll	40.856165752751984, 14.263593152756295
**C7**	Unlicensed Street Vendor	Via Giovanni Antonio Campano	40.89426512066759, 14.22739930989755
**C8**	Fish Market	Via Cavalleggeri D'Aosta	40.81686034236916, 14.18787852576902
**C9**	Unlicensed Street Vendor	Rotonda di Agnano	40.81812893794484, 14.174106045258094
**C10**	Unlicensed Street Vendor	Via Montagna Spaccata	40.856289363662064, 14.157686288997436

Table S2. Concentration of *E. coli* in the ten seized samples (from C1 to C10) and expressed in MPN/100 g

ID	*E. coli* MPN/100g
**C1**	7900
**C2**	11000
**C3**	9300
**C4**	4200
**C5**	14000
**C6**	24000
**C7**	9200
**C8**	3000
**C9**	920
**C10**	11000

Table S3. Percentage of correspondence with reference spectra for each seized sample

Seizure	Phlegraean area	Greece	Spain
**C1**	100	0	0
**C2**	6.5	59	34.5
**C3**	93	0	7
**C4**	58	11	31
**C5**	0	61.5	38.5
**C6**	92.5	3	4.5
**C7**	91	0	9
**C8**	71.5	0	28.5
**C9**	8	82	10
**C10**	83.5	2.5	14

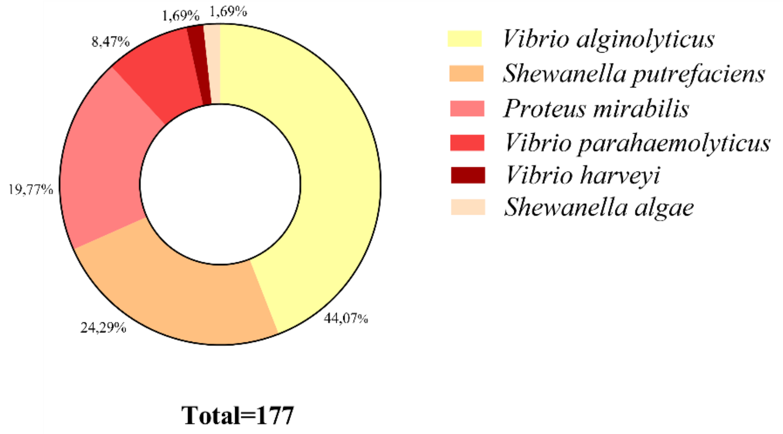

Figure S1: Percentages of bacterial species isolated from 10 batches of mussels.

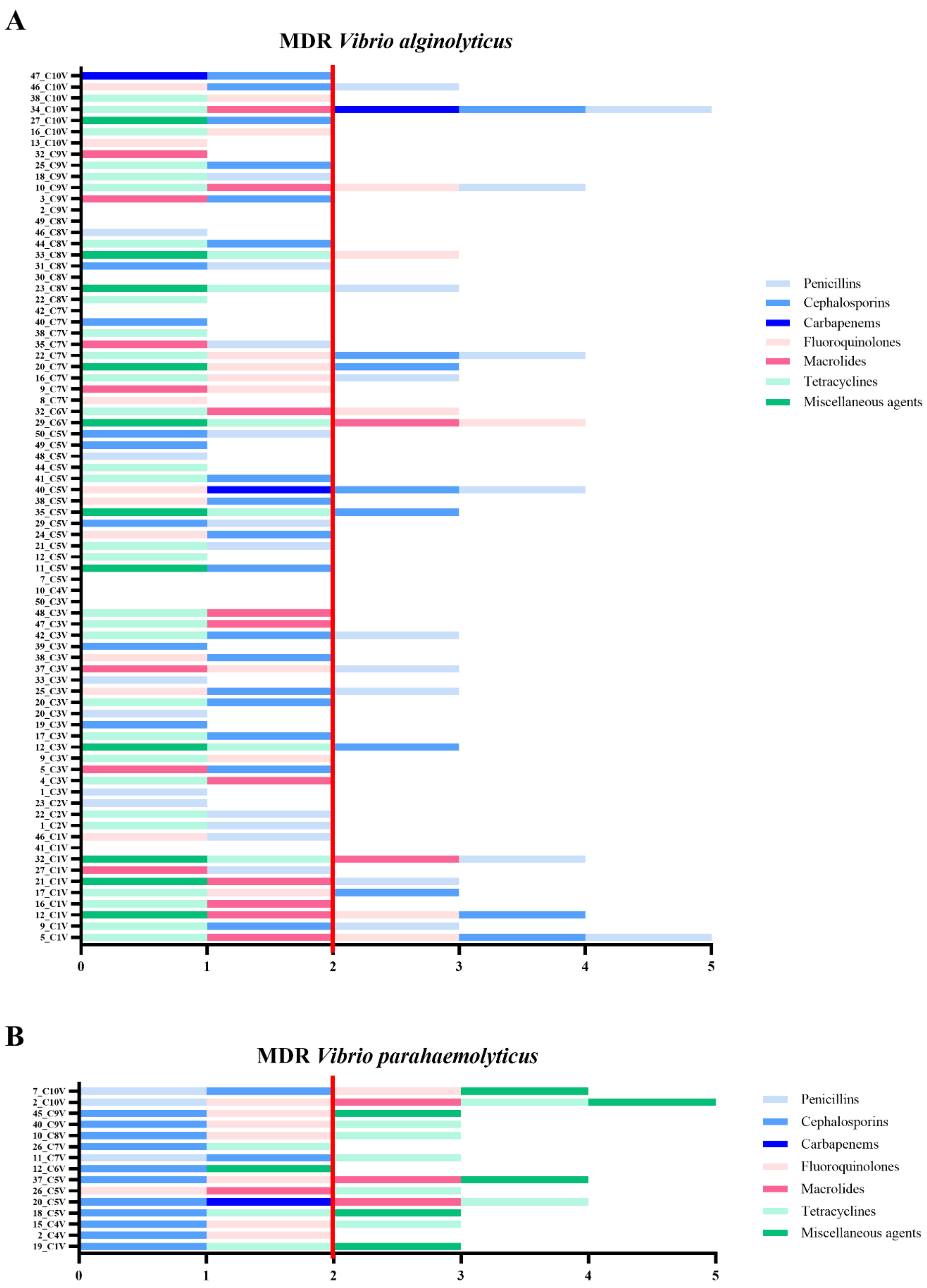

Figure S2. Representation of resistance profiles of each *Vibrio* bacterial isolate in relation to antibiotic classes. Each horizontal bar represents a single isolate and the antibiotic classes to which it showed phenotypic resistance. Antibiotic classes are color-coded. The red vertical line indicates the limit beyond which the isolates are considered MDR, as they are resistant to three or more different antibiotic classes.

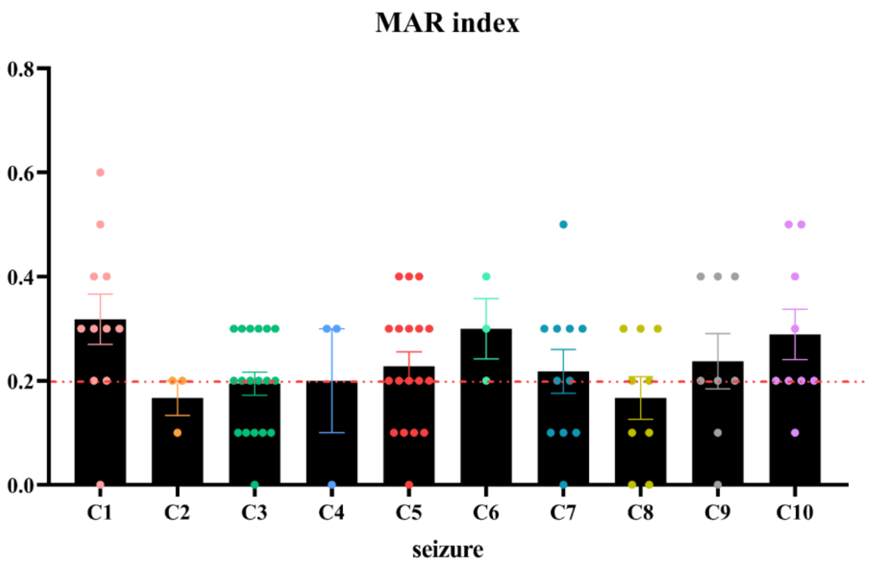

Figure S3. Average MAR index values (± error standard) calculated for *Vibrio* strains belonging to each single seized sample
